# Antioxidant Activities of Essential Oils and Their Major Components in Scavenging Free Radicals, Inhibiting Lipid Oxidation and Reducing Cellular Oxidative Stress

**DOI:** 10.3390/molecules28114559

**Published:** 2023-06-05

**Authors:** Xiaohua Chen, Shufeng Shang, Fei Yan, Hai Jiang, Guanjie Zhao, Shan Tian, Rui Chen, Dejing Chen, Yafeng Dang

**Affiliations:** 1 Shaanxi Key Laboratory of Bioresources Qinling-Bashan Mountains Bioresources Comprehensive Development CIC, School of Biological Science and Engineering, Shaanxi University of Technology, Hanzhong 723000, China; shangshf@snut.edu.cn (S.S.); yanfei@snut.edu.cn (F.Y.); jianghai0318@163.com (H.J.); zhaoguanjie@snut.edu.cn (G.Z.); rchen0411@163.com (R.C.); cdjslg@126.com (D.C.); 2 Department of Medical Oncology of 3201 Hospital, Hanzhong 723000, China; tianshan0920@163.com; 3 Inspection and Testing Center of Food and Drug, Hanzhong 723000, China; dangyafeng888@163.com

**Keywords:** essential oil, fish oil, red blood cell, antioxidant, eugenol, thymol

## Abstract

**Highlights:**

**What are the main findings?**

- The essential oils from cinnamon, thyme, clove and their main components, eugenol and thymol, exhibited the highest antioxidant activity in the FOE and RBC systems.
- It was found that the antioxidant activity of essential oils was positively correlated to the content of eugenol and thymol.

**What is the implication of the main finding?**

- Valuable evidence for the potential use of essential oils to improve human health was provided.
- Cinnamon, clove and thyme oils are expected to prevent fish oil oxidation.

**Abstract:**

Antioxidant activities of five essential oils (cinnamon, thyme, clove, lavender and peppermint oils) and their major components (eugenol, thymol, linalool, and menthol) were evaluated on scavenging DPPH (2,2-diphenyl-1 picrylhydrazyl) free radicals, inhibiting polyunsaturated fatty acid oxidation in fish oil emulsion (FOE), and reducing oxidative stress in human red blood cells (RBC). The essential oils from cinnamon, thyme, clove and their main components, eugenol and thymol, exhibited the highest antioxidant activity in the FOE and RBC systems. It was found that the antioxidant activity of essential oils was positively correlated to the content of eugenol and thymol, while lavender and peppermint oils and their main components, linalool and menthol, had very low antioxidant activity. Compared with scavenging DPPH free radical activity, the antioxidant activity in FOE and RBC systems could better reflect the actual antioxidant potential of essential oil in preventing lipid oxidation and reducing oxidative stress in biological system.

## 1. Introduction

Essential oil is a complex mixture mainly consisting of terpenoids and some other non- terpenoids such as phenylpropane. They are widely used in food condiment, perfumes, cosmetics, and medicines [1]. Essential oil has health benefit of preventing chronic and infectious diseases, improve appetite and digestion, and regulate the characteristics of intestinal flora [2]. For example, some essential oils can improve the immune status through reducing lymphocyte proliferation and phagocytosis rate and increasing serum level [3]. Recently, antioxidant potential of essential oil has been intensively studied and suggested to play an important role in anti-diabetic, anti-inflammatory, anti-hyperlipidemic, anti-hypertensive, anti-inflammatory as well as prevention of other chronic diseases because of these diseases usually result from the cellular oxidative damage caused by free radicals [4,5,6].

However, most methods for evaluation of antioxidant activity of essential oils are based on redox reaction chemical assays. The results may not reflect the activity in a biological system which involves cell membrane permeability, cellular substrates, distribution and delivery of the antioxidant between aqueous and lipid phases in the biological system [7,8]. For example, antioxidant butylated hydroxyanisole (BHA) and resveratrol (RSV) showed low reaction rates in the DPPH assay, but had high reaction rates in stabilizing the lipid vulnerable to oxidation in an emulsion system [9]. Actually, biological fluid, for example, blood, is an emulsion consisting of water, lipid micelles, proteins and other components [10]. Therefore, the results from a lipid emulsion model may closely reflect the real antioxidant potential of antioxidants in a human blood environment. Additionally, it was reported that phenolics, tamarixetin, myricetin, and kaempferol showed the highest activity in the human red blood cell system, while the compounds showed low activity in chemical oxygen radical absorbance capacity (ORAC) assay [11]. A synergistic effect between pomegranate and apple extracts was observed in the human red blood cell system, but not in the ORAC assay. The oil emulsion and red blood cell models mentioned above have been extensively used to evaluate the antioxidant potential of natural product extracts [9,10,11,12]. To the best of our knowledge, there were few reports on the antioxidant activity of essential oils in human blood.

Meanwhile in consideration of human health and safety, artificial antioxidants and antibiotics used in foods and medicines have been strictly regulated. Essential oil is a natural extract and has been recommended as one of the alternatives for food preservation to stabilize food lipids and prevent food spoilage [13]. In this study, a group of essential oils (cinnamon, thyme, clove, lavender and peppermint) and their main components (thymol, eugenol, linalool and menthol) were selected to evaluate their antioxidant activities, using a fish oil-in-water emulsion system and the human red blood cell system. Additionally, the results were compared to the antioxidant activity in the DPPH assay. The results of this study could be helpful to understand the antioxidant activity of essential oil in preventing lipid oxidation and reducing cellular oxidative stress. Also, the finding of this study could reveal the differences of antioxidant activity between different essential oils and their major components.

## 2. Results and Discussion

### 2.1. Antioxidant Activity of Scavenging DPPH free Radical

**Figure 1:**
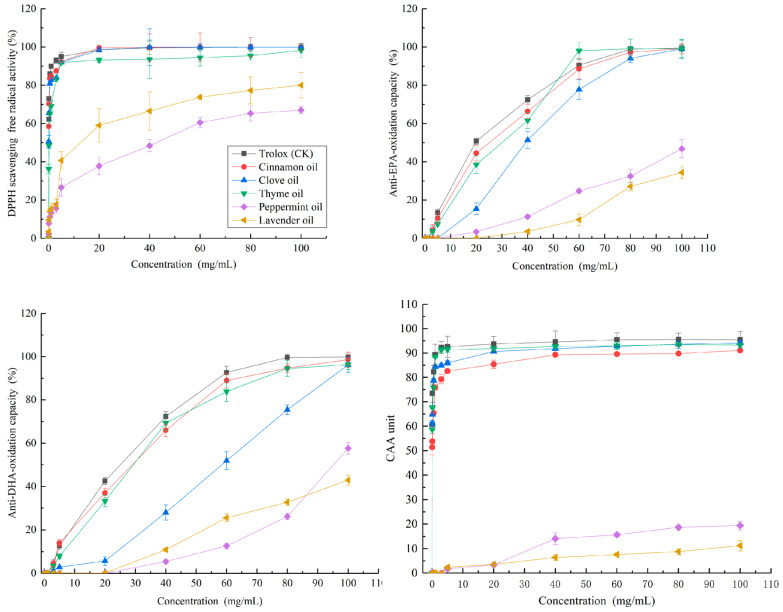
The scavenging DPPH free radical activity, anti-EPA and DHA-oxidation capability, and cellular antioxidant activity of Trolox, cinnamon, clove, thyme, lavender, and peppermint essential oils at different concentrations.

**Figure 2:**
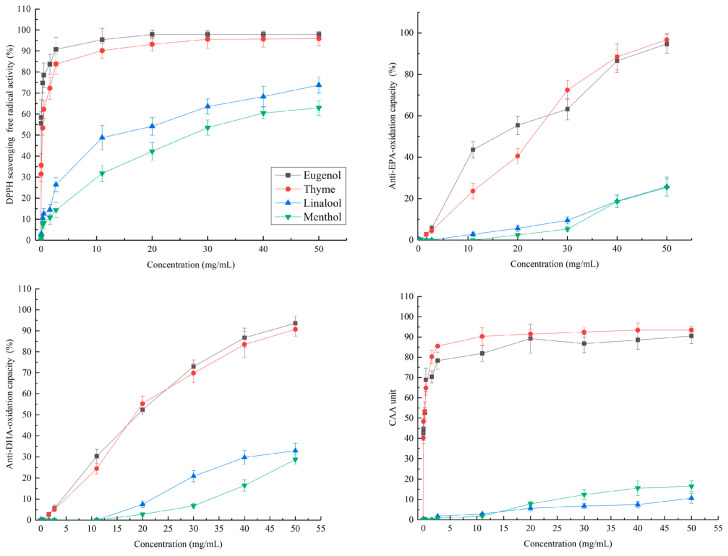
The scavenging DPPH free radical activity, anti-EPA and DHA-oxidation capability, and cellular antioxidant activity of eugenol, thymol, linalool, and menthol at different concentrations.

The major compounds in the five essential oils are listed in Table 1. Both cinnamon and clove essential oils contained high levels of eugenol (547.0 mg/mL and 517.8 mg/mL, respectively), while thyme essential oil was rich in thymol (304.0 mg/mL). Lavender essential oil had high level of linalool (307.5 mg/mL). Menthol (383.0 mg/mL) was the major component in peppermint essential oil. In addition, cinnamaldehyde (104.3 mg/mL) in cinnamon oil, β-caryophyllene (98.6 mg/mL) in thyme oil, limonene (127.0 mg/mL) in clove oil, linalyl acetate (115.4 mg/mL) in lavender oil, and menthone (93.2 mg/mL) in peppermint oil also exhibited moderate levels. The antioxidant activities of scavenging DPPH free radical of cinnamon, clove, thyme, lavender and peppermint essential oils and their main components are shown in Figure 1 and Figure 2. As half-maximal effective concentration EC_50_ value is an important index to evaluate the antioxidant potential, the EC_50_ values of cinnamon, clove, thyme, lavender and peppermint essential oils and their main components were used to compare their antioxidant activities (Table 2). A dose-dependent increase of scavenging DPPH free radical activity was observed in all essential oils. The highest scavenging DPPH free radical activity was recorded by essential oils of cinnamon (EC_50_ = 0.03 ± 0.00 mg/mL) and clove (EC_50_ = 0.05 ± 0.01 mg/mL), which was similar to that of the positive control Trolox. Correlation analysis showed that there was a significant correlation between the antioxidant activity of eugenol and that of cinnamon oil (R^2^ = 0.986) and clove oil (R^2^ = 0.990). It indicated that the antioxidant activity of cinnamon and clove essential oil was mainly from eugenol. Moderate activity was detected for thyme oil (EC_50_ = 0.14 ± 0.05 mg/mL) and its main constituent thymol (0.17 ± 0.06 mg/mL). A significant correlation of antioxidant activity between thyme oil and thymol (R^2^ = 0.975) indicated that the antioxidant activity of thyme oil was mainly due to thymol. The essential oil of lavender and peppermint showed weak activity in this test with EC_50_ value of 12.1 mg/mL and 33.9 mg/mL respectively, as well as for the corresponding main compounds. Similar results were also reported in previous studies. For example, Alfikri et al. [14] observed that the essential oil of clove (eugenol acetate/eugenol chemotype) exhibited high scavenging ability of DPPH radicals (EC_50_ = 0.015~0.18 mg/mL). Additionally, Chaieb et al. [15] and Gedikoğlu [16] reported that the EC_50_ value for the DPPH scavenging activity of clove and thyme oils was 0.04 mg/mL and 0.15mg/mL, respectively. Wu et al. [17] found that the EC_50_ value for the DPPH scavenging activity of peppermint oil was about 70 mg/mL. The high EC_50_ value (6~51 mg/mL) for the DPPH scavenging activity of lavender oil has also been reported by Silva et al. [18] and Martucci et al. [19]. Lemos et al. [20] reported that thymol had a significantly higher level of free radical scavenging activity than alcohols and terpenoids, and a moderate degree of correlation was observed between thymol and thyme oil in antioxidant activity. Terenina et al. [21] found that carvacrol and thymol were the main antioxidant compounds of oregano oil. Compared with phenolic compounds, the menthol and linalool presented a low antioxidant effectiveness [22,23]. The hydroxyl groups linked with a benzene ring plays an important role in the antioxidant activity of compounds, the groups could donate hydrogen to stabilize free radicals [9]. Therefore, the high antioxidant activity of eugenol and thymol is mainly attributed to the phenolic groups in their chemical structure.

### 2.2. Antioxidant Activity of Essential Oil in Fish Oil-in-Water Emulsion System

Previous studies have shown that the retention rate of eicosapentaenoic acid (EPA) and docosahexaenoic acid (DHA) in fish oil-in-water emulsion are considered to reflect the antioxidant potential of antioxidants in biological systems [9]. Therefore, in this study, the antioxidant potential of essential oils from cinnamon, thyme, clove, lavender and peppermint in biological systems was evaluated using the fish oil-in-water emulsion model.

The essential oils and main compounds tested showed the phenol- rich essential oils (cinnamon, thyme, and clove oils) were the most active, with EC_50_ ranging from 20.8 to 32.4 mg/mL for anti-EPA oxidant and from 21.4 to 49.2 mg/mL for anti-DHA oxidant experiments, respectively (Table 2). Its main constituents, eugenol and thymol also showed an important activity, with EC_50_ ranging from 15.3 to 18.3 mg/mL in anti-EPA oxidant experiment and from 16.4 to 17.6 mg/mL in anti-DHA oxidant experiment. Lavender and peppermint oils showed weak activity, which produced retention rate of 34% and 47% respectively at the highest concentration tested in anti-EPA oxidant experiment, and 58% and 43% respectively in anti-DHA oxidant experiment, respectively (Figure 1 and Figure 2). A significant correlation of antioxidant activity between the essential oils and their main compounds (R^2^ = 0.931~0.995) indicated the antioxidant activity of the essential oils in biological systems was mainly due to eugenol, thymol, linalool, and menthol, respectively. According to Amorati et al. [24], the activity of eugenol and thymol was related to slowing down the peroxidation of unsaturated lipids by binding H atoms in phenolic hydroxyl groups to peroxyl radicals, and the activity of linalool and menthol was attributed to co-oxidation with substrates, resulting in rapid self-termination and cross termination of oxidation chains [25,26,27,28].

Although similar trends were shown in the fish oil-in-water emulsion model and DPPH assay, the minimum response concentration of the essential oil in the two systems was significantly different (Figure 1 and Figure 2). For example, the minimum response concentration was 0.05 mg/mL in the DPPH assay, while it was 3 mg/mL for cinnamon, thyme and clove oils, and 40 mg/mL for peppermint and lavender essential oils in the emulsifying system, with a difference of 60–800 times. Similarly, the EC_50_ of cinnamon, clove, thyme, lavender and peppermint oils was 0.03–33.9 mg/mL in the DPPH assay, while it was 20.8–203.8 mg/mL in the emulsion model, with a difference of 10–600 times (Table 2). The results demonstrated the difference of the performances of the essential oils in scavenging DPPH free radical and anti-lipid-oxidation measurements. The essential oils exhibited higher scavenging DPPH free radical activity, while they had weaker performance in inhibiting lipid oxidation in the oil emulsion, which might be related to the hydrophobic characteristic of essential oil [7]. Essential oils could readily dissolve in the DPPH system, and react with DPPH rapidly in the DPPH assay, while the interface between essential oil and fish oil was difficult to contact effectively due to the obstruction of water in the fish oil-in-water emulsion system, which might make the antioxidant activity of essential oil in the emulsion system weaker than that in DPPH system. In addition, the volatility of essential oil may also be one of the important factors affecting its antioxidant activity in the emulsion system. For example, the essential oil in the fish oil-in-water emulsion system can easily diffuse to the surface of the emulsion system due to its high volatility, while the fish oil is homogeneous inside the emulsion system, which made it more difficult for the essential oil to effectively contact with the fish oil. In any way, we agree with [29] that the fish oil-in-water emulsion system model may be more predictive of the antioxidant activity of essential oils in biological systems than DPPH analysis, especially at low concentrations.

EPA and DHA can enhance human immunity, reduce severity of disease, relieve childhood depression, and improve cognitive ability [30]. The oxidation of EPA and DHA in fish oil usually causes undesirable fishy and pungent odors, low nutritional quality and safety in fish oil-containing foods [31]. Among them, the off-flavors are the most serious problem that limits the addition of fish oil to general food and beverage products, and acrolein and vinyl ketone are considered to be the major contributors to the off-flavors [32,33]. Although a lot of studies have been done on the formation mechanism of off-flavors during EPA and DHA oxidation, the prevention of volatile compounds formed is still significantly challenging [31]. In the study, the essential oils of cinnamon, clove, thyme, peppermint and lavender showed considerable anti-EPA and DHA oxidation ability. Moreover, the pleasant aroma released by the essential oils can effectively cover these off-flavors, which provided another perspective for solving the problem.

### 2.3. Cellular Antioxidant Activity in Red Blood Cells System (CAA-RBC)

A CAA-RBC assay was used to investigated the free-radical scavenging capacity of cinnamon, clove, thyme, lavender, and peppermint essential oils in red blood cells. Results on the activity of essential oils and its compounds were shown in Table 2 and Figure 1 and Figure 2. Among the essential oils, clove and thyme essential oils gave the lowest EC_50_ values of 0.22 mg/mL and 0.23 mg/mL respectively, similar to the positive control Trolox (0.25 mg/mL). This result highlights the capacity of clove and thyme oils to prevent cell damage by free radicals, which was not previously reported in the literature. The cinnamon oil also exhibited considerable activity with EC_50_ of 0.47 mg/mL, while the activity of lavender and peppermint oils was weak with EC_50_ of 305.9 mg/mL and 761.2 mg/mL, respectively. Correlation analysis showed that the AAPH inhibitory activity of clove, cinnamon and thyme oils could be mainly attributed to its major constituent eugenol and thymol, which gave an EC_50_ = 0.13 mg/mL and 0.12 mg/mL, respectively (R^2^ = 0.921–0.978). Although the structures of these two phenolic monoterpenes are different in the position of hydroxyl in the aromatic ring, they show very similar behavior in scavenging free radicals.

Figure 1 showed the free-radical scavenging capacity linearly increased with the essential oil concentrations. Although similar trends were also observed in DPPH assay, a significant difference in the free-radical scavenging capacity of peppermint and lavender essential oils was found between CAA-RBC and the DPPH assay. For example, the minimum concentration of free radical scavenging activity for lavender and peppermint essential oils was 0.05 mg/L in DPPH assay, while it was 5 mg/L in CAA-RBC assay, and the difference was 100 times. Together, this study gave a first insight on the cellular antioxidant activities of essential oil, providing useful information for further studies and uses of the essential oils. As shown in Table 2, clove and thyme essential oils exhibited higher cellular antioxidant activity similar to those of Trolox, with EC_50_ of 0.22 mg/mL and 0.23 mg/mL, respectively, followed by cinnamon oil with EC_50_ of 0.47 mg/mL, and the essential oil of lavender and peppermint was weaker with EC_50_ of 305.9 mg/mL and 761.2 mg/mL, respectively.

Compared with DPPH assay, the EC_50_ value of cinnamon, clove, thyme and peppermint essential oils in erythrocyte system increased by about 6-8 times, while that of lavender essential oil increased by about 60 times. Correlation analysis showed that CAA values of the thyme, lavender and peppermint essential oils were weakly correlated with DPPH results (R^2^ = 0.899, 0.635, and 0.583 for thyme, lavender and peppermint essential oil, respectively), which was consistent with the reports by Zhou et al. [10]. Red blood cells (RBC) play a key role in reducing oxidative stress in the vascular system, and its antioxidant capacity is relevant in the body [34].Therefore, the free radical scavenging activity of the essential oils in erythrocyte system reflected its actual antioxidant potential in human body. CAA-RBC assay is a useful tool to evaluate the intracellular biological activity of phytochemicals. Our results showed that these essential oils with significant antioxidant activity in vitro also have considerable antioxidant activity in human red blood cells. RBC is a cell without nucleus and mitochondria, which excludes both the interfering contribution of oxygen radicals on gene transcription and the production of mitochondrial reactive oxygen species [34]. Thus, the weaker free radical scavenging activity of the essential oils in human erythrocyte system than that in DPPH system might mainly due to the obstruction of cell membrane to essential oil [11].

## 3. Materials and Methods

### 3.1. Chemicals

The essential oils of cinnamon, thyme, clove, lavender, and peppermint were obtained from the local aromatic plant laboratory (Hanzhong, Shaanxi, China). Menhaden fish oil was purchased from Blackmores Ltd. (Sydney, Australia). Tween 20, cinnamaldehyde, eugenol, thymol, linalool, and menthol were obtained from ANPEL Laboratory Technologies Inc. (Shanghai, China). The 2,2-diphenyl-l-picrylhydrazyl (DPPH) methyl ester, Eicosapentaenoic acid (EPA) methyl ester, docosahexaenoic acid (DHA) methyl ester, 2′,7′-dichlorofluorescein diacetate (DCFH-DA), 2,2′-azobis (2-amidinopropane) dihydrochloride (AAPH), Trolox, hexane, methanol, and anhydrous ethanol were obtained from J&K Scientific Ltd. (Beijing, China). All other common analytical reagents used in the study were purchased from Sinopharm Chemical Reagents Co., Ltd. (Shanghai, China).

### 3.2. Identification and Quantification of Main Components in Essential Oil by GC-MS and GC-FID

The samples of components in essential oil were analyzed using a Thermo Trace gas chromatography (GC), coupled with a Thermo ITQ900 Mass Spectrometer (MS) Detector (Thermo Scientific, Waltham, MA, USA) and a DB-5MS (30 m × 0.25 mm i.d. × 0.25 μm film thickness) column. The electron ionization energy was 70 eV. The ion source and transfer line temperatures were set at 250 and 230 °C, respectively. The scan range of the MS was set at 40–400 *m*/*z* with a scan rate of 1 scan/s. Helium was used as a carrier gas at a constant flow of 1 mL/min. The injector temperature of GC was 200 °C. The GC oven temperature was held at 60 °C for 3 min, then ramped to 180 °C at a rate of 3 °C/min. The injected volume was 1 μL with split ratio 30:1. The compounds were identified by their authentic standards, a mass spectrum in the NIST 2014 library, and retention indices calculated using the retention times of alkanes (C9–C27). The quantification of each compound was carried out using an external standard method in a Shimadzu 2010 plus GC system (Shimadzu, Tokyo, Japan) with a flame ionization detector. The temperature of FID was 250 °C. The other GC operation conditions were the same as the GC-MS method above.

### 3.3. Determination of Antioxidant Activity Using DPPH Assay

The radical scavenging activity of essential oils or each of their main compounds was evaluated using a modified DPPH assay as described by [9]. The free radical DPPH was dissolved in methanol and prepared to be 0.1 M solution. A sample solution (0.1 mL) with different concentrations was reacted with 0.1 mL of DPPH solution in the well of a 96-well plate for 30 min at 25 °C in the dark. Blank sample was prepared by mixing methanol (0.1 mL) with the prepared DPPH solution. Trolox was used as positive control. The absorbance of each reaction solution was measured at wavelength 517 nm. The scavenging DPPH free radical percentage was calculated as follows:

Scavenging DPPH free radical percentage (%) = [(A_0_ − A_1_)/A_0_] × 100%

where: A_0_ is absorbance of blank sample, A_1_ is the absorbance of test solution

### 3.4. Determination of Antioxidant Activity Using Fish Oil Emulsion System

A fish oil emulsion consisting of 10 mg/mL of menhaden fish oil and 1.0 mg/mL of Tween 20 in phosphate buffer (pH 7.0) was prepared by using the procedure in the study of Zhang et al. [9]. Sample solution (0.5 mL) was mixed with 15.0 mL of the fish oil emulsion in a 50.0 mL vial and then incubated at 37 °C for 72 h with shaking. The extraction of EPA (eicosapentaenoic acid) and DHA (docosehexaenoic acid) was carried out by using the method reported by Zhang et al. [9]. Trolox was used as positive control. The concentrations of EPA and DHA in each fish oil emulsion at 0 and 72 h were determined by using a Shimadzu GC2010 plus-flame ionization detection (FID) system, coupled with external standard method. The GC column was InertCap-Wax (30 m × 0.25 mm i.d. × 0.25 μm film thickness) (Shimadzu, Japan). Nitrogen (purity = 99.999%) was used as a carrier gas at a constant flow of 1 mL/min. The injector temperature was 200 °C. The temperature program was performed based on the method described in the study of Chen et al. [35], namely the GC oven temperature was held at 40 °C for 5 min, then ramped to 220 °C at a rate of 3 °C/min and held for 5 min.

The antioxidation activity was calculated by the formula below:

Anti-fish oil-oxidation activity (%) = (C_t_ − C_0_) × 100

where C_0_ is the concentration (μg/mL) of EPA or DHA at 0 h; C_t_ is the concentration (μg/mL) of EPA or DHA at 72 h in the same emulsion.

### 3.5. Determination of Antioxidant Activity Using Red Blood Cell System

The antioxidant activity against oxidative stress red blood cell was carried out based on the method described by Blasa et al. [11] and Frassinetti et al. [36]. The human blood from healthy volunteers was collected by the local hospital according to the WS/T 661-2020 (Guidelines of venous blood specimen collection). The red blood cell solution was prepared in phosphate buffer saline (PBS, pH 7.4), with the number of red blood cells approximately 10^5^ cells/mL. The prepared solution (250 μL) was incubated at 37 ℃ for 1 h with 150.0 μL of DCFDA fluorogenic dye (25 μM) and 100.0 μL of sample solution in a centrifuge tube. Trolox solution was used as positive control, while PBS solution was used as blank. After incubation, the cells were washed twice with PBS and then suspended in 500.0 μL of PBS. After 180.0 µL of the cell suspension was transferred in a 96-well microplate, AAPH solution (20.0 µL, 1.2 mM) was added to each well. The fluorescence intensity of each well was read at 485 nm excitation and 535 nm emission by using an Infinite M200 PRO plate reader (Tecan, Männedorf, Switzerland). The cellular antioxidant activity was expressed as CAA (cellular antioxidant activity) was calculated using the following formula reported by Wolfe and Liu [37]:

CAA unit = 100 − (∫SA⁄∫CA) × 100

where: ∫SA was the integrated area under the sample curve, ∫CA was the integrated area under the control curve.

### 3.6. Statistical Analysis

Each measurement was repeated in triplicate. Data was expressed as means with standard deviation. The EC_50_ value and significant difference (*p* < 0.05) were calculated by regression analysis and one-way analysis of variance (ANOVA), respectively, using the SPSS v21.0 program (SPSS Inc., Chicago, IL, USA). The correlation analysis was performed by Microsoft Excel (Redmond, WA, USA).

## 4. Conclusions

The present study investigated the antioxidant potential in fish oil-in-water emulsion system and free radical scavenging activity in human erythrocyte system for cinnamon, thyme, clove, lavender and peppermint essential oils and their main constituents, and also discussed the effects of cell membrane permeability, and human blood emulsification environment on antioxidant activity. The phenol-rich essential oils (cinnamon, thyme, and clove oils) and their main constituents showed the best antioxidant profile in fish oil-in-water emulsion and human erythrocyte systems, close to the positive controls, which implies good antioxidant potential in biological systems. Correlation analysis showed that the high antioxidant activity of these essential oils in biological system was mainly due to the high content of eugenol and thymol in essential oils. Non phenolic essential oils (lavender and peppermint oils) show very weak antioxidant activity, which is related to the weak antioxidant behavior of linalool and menthol in essential oil. Compared with a non-biological system (DPPH system), the antioxidant activity of phenolic and non-phenolic essential oils in the biological system (fish oil-in-water emulsion and human erythrocyte systems) decreased significantly, which might be closely related to the distribution proportion of essential oils at the oil-water interface and membrane permeability. Although most of the synthetic antioxidants had a higher antioxidant capacity, the risks from their toxicity and potential side effects on the body has led people to pay more attention to natural antioxidants, with a particular interest in those that are frequently consumed by people. This study provides the first insight into the antioxidant activity of essential oils in human red blood cells and in the blood emulsification environment. The results of the study provided valuable evidence for the potential use of essential oil as a good source of natural antioxidants for improving human health.

## Figures and Tables

**Table 1:** Chemical names and concentrations of main compounds in essential oil of cinnamon, clove, thyme, lavender, and peppermint (RI: retention index; *nd*: no detection; *tr*: traces (<0.1); *^a^*: the relative concentration of the compound was obtained by an external standard method; *: significant difference at *p* < 0.05).

RI	Compound	Cinnamon	Clove	Thyme	Lavender	Peppermint
		Relative Concentration (mg/mL) *^a^*				
2235	Eugenol	547 ± 10.4 *	517.8 ± 8.6 *	5.0 ± 0.8	*tr*	*nd*
2256	Thymol	*tr*	*tr*	304 ± 2.8 *	*nd*	*tr*
1629	Linalool	5.2 ± 0.68	*nd*	*tr*	307.5 ± 7.3 *	*tr*
1715	Menthol	*nd*	*nd*	*nd*	*nd*	383 ± 16.2 *

**Table 2:** The EC_50_ values of scavenging DPPH free radical activity, anti-lipid-oxidation capability, and CAA-RBC of the cinnamon, clove, thyme, lavender and peppermint essential oils and their main compounds (*^a^*^–^*^d^*: significant difference at *p* < 0.05).

	EC_50_ (mg/mL)		
	DPPH Assay	Anti-EPA/DHA Oxidation Capability	CAA-RBC
Trolox (CK)	0.04 ± 0.01 *^a^*	18.6 ± 1.15 *^a^/*20.2 ± 2.08 *^a^*	0.25 ± 0.14 *^a^*
Cinnamon oil	0.03 ± 0.00 *^a^*	20.8 ± 3.27 *^a^/*21.4 ± 1.21 *^a^*	0.47 ± 0.12 *^b^*
Clove oil	0.05 ± 0.01 *^a^*	32.4 ± 2.23 *^b^/*49.2 ± 7.63 *^b^*	0.23 ± 0.03 *^a^*
Thyme oil	0.14 ± 0.05 *^b^*	21.7 ± 1.87 *^a^/*24.3 ± 3.25 *^a^*	0.22 ± 0.06 *^a^*
Lavender oil	12.1 ± 2.16 *^c^*	125.8 ± 23.54 *^c^/*132.9 ± 17.68 *^d^*	761.2 ± 4.25 *^d^*
Peppermint oil	33.9 ± 5.64 *^d^*	203.8 ±15.55 *^d^/*144.2 ± 9.86 *^d^*	305.9 ± 13.42 *^c^*
Eugenol	0.26 ± 0.01 *^b^*	15.3 ± 3.16 *^a^/*16.4 ± 3.26 *^a^*	0.13 ± 0.22 *^b^*
Thymol	0.17 ± 0.06 *^b^*	18.3 ± 1.57 *^a^/*17.6 ± 3.16 *^a^*	0.12 ± 0.02 *^a^*
Linalool	12.8 ± 1.28 *^c^*	104.5 ± 13.33 *^c^/*79.6 ± 8.62 *^c^*	513.9 ± 9.25 *^d^*
Menthol	26.6 ± 3.52 *^d^*	126.5 ±10.25 *^c^*/118.5 ± 12.34 *^d^*	150.8 ± 8.56 *^c^*

## Data Availability

Not applicable.

## References

[1] Diniz do Nascimento L., Barbosa de Moraes A.A., Santana da Costa K., Pereira Galúcio J.M., Taube P.S., Leal Costa C.M., Neves Cruz J., de Aguiar Andrade E.H., Guerreiro de Faria L.J. (2020). Bioactive natural compounds and antioxidant activity of essential oils from spice plants: New findings and potential applications. Biomolecules.

[2] Franz C., Baser K.H.C., Windisch W. (2009). Essential oils and aromatic plants in animal feeding—A European perspective. A review. Flavour Fragr. J..

[3] Li S.Y., Ru Y.J., Liu M., Xu B., Péron A., Shi X.G. (2021). The effect of essential oils on performance, immunity and gut microbial population in weaner pigs. Lives Sci..

[4] Miguel M.G. (2010). Antioxidant and anti-Inflammatory activities of essential oils: A short review. Molecules.

[5] Amorati R., Foti M.C., Valgimigli L. (2013). Antioxidant activity of essential oils. J. Agric. Food Chem..

[6] Cheli F., Baldi A. (2011). Nutrition-based health: Cell-based bioassays for food antioxidant activity evaluation. J. Food Sci..

[7] Frankel E.N., Meyer A.S. (2000). The problems of using one-dimensional methods to evaluate multifunctional food and biological antioxidants. J. Sci. Food Agric..

[8] Liu R.H., Finley J. (2005). Potential cell culture models for antioxidant research. J. Agric. Food Chem..

[9] Zhang Y., Shen Y., Zhu Y., Xu Z. (2015). Assessment of the correlations between reducing power, scavenging DPPH activity and anti-lipid-oxidation capability of phenolic antioxidants. LWT—Food Sci. Technol..

[10] Zhou Q., Lu W., Niu Y., Liu J., Zhang X., Gao B., Akoh C.C., Shi H., Yu L. (2013). Identification and quantification of phytochemical composition and anti-inflammatory, cellular antioxidant, and radical scavenging activities of 12 Plantago species. J. Agric. Food Chem..

[11] Blasa M., Angelino D., Gennari L., Ninfali P. (2011). The cellular antioxidant activity in red blood cells (CAA-RBC): A new approach to bioavailability and synergy of phytochemicals and botanical extracts. Food Chem..

[12] López-Alarcón C., Denicola A. (2013). Evaluating the antioxidant capacity of natural products: A review on chemical and cellular-based assays. Anal. Chim. Acta.

[13] Ordóñez G., Llopis N., Peñalver P. (2008). Efficacy of eugenol against a Salmonella enterica serovar enteritidis experimental infection in commercial layers in production. J. Appl. Poult. Res..

[14] Alfikri D.N., Pujiarti R., Wibisono M.G., Hardiyanto E.B. (2020). Yield, quality, and antioxidant activity of clove (*Syzygium aromaticum* L.) bud oil at the different phenological stages in young and mature trees. Scientifica.

[15] Chaieb K., Zmantar T., Ksouri R., Hajlaoui H., Mahdouani K., Abdelly C., Bakhrouf A. (2007). Antioxidant properties of the essential oil of Eugenia caryophyllata and its antifungal activity against a large number of clinical candida species. Mycoses.

[16] Gedikoğlu A., Sökmen M., Çivit A. (2019). Evaluation of *Thymus vulgaris* and *Thymbra spicata* essential oils and plant extracts for chemical composition, antioxidant, and antimicrobial properties. Food Sci. Nutr..

[17] Wu Z., Tan B., Liu Y., Dunn J., Guerola P.M., Tortajada M., Cao Z., Ji P. (2019). Chemical composition and antioxidant properties of essential oils from peppermint, native Spearmint and scotch spearmint. Molecules.

[18] DA Silva G.L., Luft C., Lunardelli A., Amaral R.H., Melo D.A.D.S., Donadio M.V., Nunes F.B., DE Azambuja M.S., Santana J.C., Moraes C.M. (2015). Antioxidant, analgesic and anti-inflammatory effects of lavender essential oil. Ann. Braz. Acad. Sci..

[19] Martucci J.F., Gende L.B., Neira L.M., Ruseckaite R.A. (2015). Oregano and lavender essential oils as antioxidant and antimicrobial additives of biogenic gelatin films. Ind. Crop. Prod..

[20] Lemos M.F., Lemos M.F., Pacheco H.P., Guimarães A.C., Fronza M., Endringer D.C., Scherer R. (2017). Seasonal variation affects the composition and antibacterial and antioxidant activities of *Thymus vulgaris*. Ind. Crop. Prod..

[21] Terenina M.B., Misharina T.A.N., Krikunova I., Alinkina E.S. (2011). Oregano essential oil as an inhibitor of higher fatty acid oxidation. Appl. Biochem. Microbiol..

[22] Ruberto G., Baratta M.T. (2000). Antioxidant activity of selected essential oil components in two lipid model systems. Food Chem..

[23] Liu K., Chen Q., Liu Y., Zhou X., Wang X. (2012). Isolation and biological activities of decanal, linalool, valencene, and octanal from sweet orange oil. J. Food Sci..

[24] Amorati R., Baschieri A., Morroni G., Gambino R., Valgimigli L. (2016). Peroxyl radical reactions in water solution: A gym for proton-coupled electron-transfer theories. Food Chem..

[25] Sacchetti G., Maietti S., Muzzoli M., Scaglianti M., Manfredini S., Radice M., Bruni R. (2005). Comparative evaluation of 11 essential oils of different origin as functional antioxidants, antiradicals and antimicrobials in foods. Food Chem..

[26] Sun Z., Wang H., Wang J., Zhou L., Yang P. (2014). Chemical composition and anti-inflammatory, Cytotoxic and antioxidant activities of essential oil from leaves of *Mentha piperita* grown in China. PLoS ONE.

[27] Baschieri A., Ajvazi M.D., Tonfack J.L.F., Valgimigli L., Amorati R. (2017). Explaining the antioxidant activity of some common non-phenolic components of essential oils. Food Chem..

[28] Kivrak Ş. (2018). Essential oil composition and antioxidant activities of eight cultivars of Lavender and *Lavandin* from western Anatolia. Ind. Crop. Prod..

[29] Zhang X., Shen Y., Prinyawiwatkul W., King J.M., Xu Z. (2015). Comparison of the activities of hydrophilic anthocyanins and lipophilic tocols in black rice bran against lipid oxidation. Food Chem..

[30] Lukiw W.J., Bazan N.G. (2008). Docosahexaenoic acid and the aging brain. J. Nutr..

[31] Kazuo M. (2019). Prevention of fish oil oxidation. J. Oleo Sci..

[32] Endo Y., Hayashi C., Yamanaka T., Takayose K., Yamaoka M., Tsuno T., Nakajima S. (2013). Linolenic acid as the main source of acrolein formed during heating of vegetable oils. J. Am. Oil Chem. Soc..

[33] Wang B., Adhikari B., Barrow C.J. (2014). Optimisation of the microencapsulation of tuna oil in gelatin-sodium hexametaphosphate using complex coacervation. Food Chem..

[34] Buehler P.W., Aalayash A.I. (2005). Redox biology of blood revisited: The role of red blood cells in maintaining circulatory reductive capacity. Antioxid. Redox Signal..

[35] Chen X., Chen D., Jiang H., Xu Z. (2019). Aroma characterization of Hanzhong black tea (*Camellia sinensis*) using solid phase extraction coupled with gas chromatography-mass spectrometry and olfactometry and sensory analysis. Food Chem..

[36] Frassinetti S., Moccia E., Caltavuturo L., Gabriele M., Longo V., Bellani L., Giorgi G., Giorgetti L. (2018). Nutraceutical potential of hemp (*Cannabis sativa* L.) seeds and sprouts. Food Chem..

[37] Wolfe K.L., Liu R.H. (2007). Cellular antioxidant activity (CAA) assay for assessing antioxidants, foods, and dietary supplements. J. Agri. Food Chem..

